# Modeling environmental variability and network formation among pastoral nomadic households: Implications for the rise of the Mongol Empire

**DOI:** 10.1371/journal.pone.0223677

**Published:** 2019-10-10

**Authors:** Daniel R. Shultz, Andre Costopoulos

**Affiliations:** 1 Department of Anthropology, McGill University, Montreal, Quebec, Canada; 2 Department of Anthropology, University of Alberta, Edmonton, Alberta, Canada; Universitat de Barcelona, SPAIN

## Abstract

We use agent-based computer simulation to test the effect of environmental conditions (available biomass/carrying capacity and environmental risk) on the development of wealth inequality and patron-client herding networks in nomadic pastoral economies. Our results show that 1) wealth inequality reaches very high levels when carrying capacity is high and risk is low, and 2) patron-client contract herding networks increase in size and duration when carrying capacity is high and risk is low. We compare empirical data from the Mongol (1206–1368 CE) and Xiongnu (209 BCE– 48 CE) empires with simulation results to develop an explanatory mechanism for the apparent correlation between nomadic empire creation and positive environmental conditions. We argue that the internal dynamics of nomadic pastoral societies are sufficient to produce high degrees of inequality and hierarchical herding networks. Nomadic empires are more likely to form during key periods of increased biomass and decreased environmental risk.

## Introduction

Climate-centric theories of nomadic empire expansion in Inner Asia have been prevalent for over a century [1–4]. According to these longstanding models, detrimental drier and/or colder conditions lessened available biomass, forcing nomads to band together for external conquest. In contrast, several recent climatological studies have linked favourable conditions, particularly an increase in available biomass, to the spread of the Mongol Empire (1206–1368 CE) [5–8].

We use agent-based simulation to test the effects of positive and negative environmental conditions on wealth inequality and patron-client networks between herding households, two variables related to nomadic empire formation. We find that in simulated pastoral populations, wealth inequality is high under most environmental conditions, and the size and duration of patron-client networks increases with available biomass.

Ethnographic and historical research indicates that patron-client herding networks resulting from an unequal distribution of wealth may be characteristic of the organization of historical nomadic societies in Inner Asia [9,10]. We hypothesize that increases in carrying capacity/biomass during periods of favourable climate would increase network size and duration, making large and successful empires or polities more likely to form and expand during these periods. Our paper thus proposes an explanatory mechanism for the relationship between a favourable environment and the initial formation of successful nomadic polities, via networks formed in response to wealth inequality.

Other models of nomadic empire formation have focused on the dependency of nomads on neighbouring sedentary civilizations, from whom nomads acquire surplus energy via trading, or raiding and conquest, allowing them to sustain unequal the hierarchies of complex polities [11,12]. Our results add to recent research challenging these theories [13,14], as we suggest that internal dynamics of pastoral nomadic economies allow the rapid emergence of wealth inequality, in line with many ethnographic reports [15,16], as well as social inequality and complexity via hierarchical networks.

Computer simulation remains an uncommon method for the study of pastoral nomadic societies in Inner Asia (but see [17–19]). This team has been developing a whole-society model of Inner Asian pastoral nomads with the aim of understanding political processes, strategies, and dynamics related to inter-group conflict and the rise and fall of tribal confederations and empires. Our simulation is narrower in scope, focusing specifically on the relationship between environmental conditions and network formation in response to wealth inequality. In this regard, it engages directly with longstanding and recent debates and research concerning climate and the creation of nomadic empires.

## Agent-based simulation

Agent-based modeling (ABM) is a type of simulation that models the behaviour of individual agents, in this case herding households. In ABM, agents exist on a landscape, and interact with each other and their environment (see [20] for the history of ABM use in archaeology). The macro-level patterns their behaviour produces manifest as emergent phenomena that can be analysed statistically. Simulations can be run many times at each combination of initial conditions, and the surprising variety of results have the potential to bridge the gap between particularistic and pattern-based explanations of human behaviour. The simulations in this paper are written in NetLogo, an open-source ABM package developed primarily for social science research [21].

The approach to ABM we follow here is about understanding abstract baseline dynamics, rather than describing the historical realities of particular regions or time periods. Instead of attempting to pinpoint temporally or spatially realistic values for parameters, we test through a wide enough range of parameter values to discover under what conditions interesting dynamics occur. The usefulness of the results for understanding specific historical situations can be determined later. Simulation can then function as a thought exercise to aid in developing interpretive ideas about actual historical, archaeological, and environmental data.

### Simulation overview

We explore the relationship between environmental conditions (available biomass and environmental risk), wealth inequality (herd size differences between households), and network formation and duration. Our simulation features independent herding households on a communal landscape, growing their herds and undergoing losses due to environmental disasters. These households are implicitly multi-generational, and thus do not die of old age. ABMs proceed according to a turn-based system of time steps. Our simulation lasts 2,000 time steps per run. We conceive of a time step as roughly similar to one year, and 2,000 years is roughly the time span in which Inner Asian nomadic polities are historically recorded as being a major political force (~300 BCE to 1700 CE). Experimentation revealed 2,000 time steps to be a sufficient length of time to capture relevant model dynamics. For more information on our choice of run time length and experimentation with longer run times, see Section D in S1 File. Households begin each simulation run with 60 animals each, and grow their herds by 10% every time step, while system carrying capacity has not been reached. Each time step they have a probability of suffering an individual environmental disaster, reducing their herd size by 50%. If carrying capacity is reached, herds stop growing until disasters lower the population below capacity. If a household’s herd descends below 60 animals, they look for a patron household whose herd size is above 800 animals. This patron transfers 60 animals to the client household, and forms a link with it. If there are no patrons, and the poor household’s herd drops below 2 animals, the household dissolves, and is removed from the simulation. We vary carrying capacity from 5,000 to 50,000 animals, in intervals of 5,000. The environmental disaster frequencies tested are 5, 10, 15, and 20%. The simulation is run 100 times at each combination of carrying capacities and disaster frequencies (thus totalling 5,000 runs). Average results from 100 runs at each combination are used for analysis, to ensure statistical robustness. For more information on the validation tests we performed to ensure 100 runs was sufficient to capture model output variance, see Section A in S1 File. Every time step, each household performs the following procedures:

1. Checks whether total animals in system is below capacity
  - If yes, grows herd by herd growth rate; proceeds to step 2
  - If no, proceeds to step 2
2. Draws a random number to check if they will suffer an environmental disaster
  - If yes, reduces herd size by 50%; proceeds to step 3
  - If no, proceeds to step 3
3. Checks if own herd size is < 60
  a If yes, and household already has a patron, patron transfers them 60 animals
  a If yes, and household has no patron, household searches for a patron
  b If patron is found, receives 60 animals from patron and a link is formed between households
  e If no patron is available, and household’s herd is < 2, household dies
4. If herd size is > 800, and another household requests to be a client, household becomes a patron
  - Transfers 60 animals to client
  - Forms link with client
5. If herd size drops < 500
  - Household loses patron status
  - Links with clients in its network are dissolved

The goal of this type of abstract ABM is to use as little real-world input data as necessary, avoiding making too many unjustifiable or inaccurate assumptions about the past. This is especially relevant when archaeological and historical data are sparse, as is the case with historical pastoral nomadic societies in Inner Asia. Assumptions are derived mainly from ethnographic analogy. If available historical and archaeological data matches well with a portion of simulation output, this adds validity to assumptions made. The dangers of equifinality remain, as with most theory-driven archaeology, but if assumptions have an ethnographic and/or historical basis, and simulation output helps fill in the sparse historical and archaeological record, then the results are as useful as theories derived by more traditional methodologies. Assumptions consist of the entities and independent variables that make up our simulation. They are described here, along with justifications for their inclusion:

#### Entities

1) Herding households, animal herds, and communal pastureland (i.e. the private ownership of herds, communal ownership of land, and the individual family household as the basic unit of the nomadic pastoral economy):

These are well established in the ethnographic literature [9,12], and remain characteristic of herding in Mongolia today [15,22]. The disastrous effects associated with forced attempts at communal herd ownership in Inner Asia during the Soviet period have been well documented [23]. Given that an extensive pastoral nomadic system requires wide dispersal of the total number of animals to ensure adequate pasture, and to prevent overgrazing, dispersed households with private herd ownership/management is a practical adaptive strategy. This provides maximum flexibility for each household to adapt to fluctuating environmental conditions in their given locality.

2) Patron-client networks:

Given private ownership of herds by households, the effects of environmental disasters are felt differentially, depending on location, as well as luck and management skill, leading to the rapid development of wealth inequality. In this regard, Cribb [9] has noted that resultant forms of social organization in nomadic pastoral societies play the role of solving discrepancies between labour and capital, or the differential herding success of households, as some have surplus animals, while others are willing labourers but have lost their herds. These latter become contract herder clients to wealthy patrons, shifting from individual household herd ownership to individual household herd management, as wealthy absentee herd owners come to own a large percentage of the total animals in the system [9]. Other social relations beyond a simple patron-client structure have been empirically observed in nomadic society, including institutionalized aristocracy [24] and communist state ownership and herd redistribution [23]. However, we are interested here in theoretical mechanisms for the initial emergence of hierarchical structures, and patron-client networks represent a simple starting point, from which more complicated social relations and political forms could emerge. In our simulation, patrons receive no benefit for sharing their animals, in the interest of simplicity. In reality, the patron-client relationship we model is essentially one of contract herding, where the patron continues to own the animals granted to clients. If we chose to model this dynamic, it would not affect network size, but would increase network duration proportionally at all parameter values. Thus, choosing a simpler model, we can still understand relative changes in network duration as a function of shifting environmental variables.

#### Independent variables

1) Carrying capacities/available biomass:

We use carrying capacity as our main environmental variable, despite the traditional non-equilibrium theory of pastoral land use. According to non-equilibrium theory, carrying capacity of pastures is never reached because natural disasters such as droughts are so frequent that herd sizes are kept low. We use carrying capacity because 1) much of modern Mongolia is not a non-equilibrium zone [25], particularly the productive central and eastern portions that formed the heartland of the Mongol and Xiongnu empires; and 2) much recent ecological research indicates that traditional non-equilibrium zones, as well as lusher equilibrium areas, suffer measurable and severe pasture degradation due to overgrazing, calling into question the main tenet of non-equilibrium theory [26]. In our simulation carrying capacity (the maximum number of animals in the system) is varied between 5,000 and 50,000, increasing in intervals of 5,000. Since our ABM simulates 100 households, this equates to an average of between 50 (very low for a herding society) and 500 animals per household, if no inequality were to develop. For comparison, in 2014 the herding economy in Mongolia consisted of 213,400 households managing a total of 51,982,600 head of livestock, or an average of 243 animals per household [27]. This equates to a capacity of slightly fewer than 25,000 in our simulation. A range of 5,000 to 50,000 was thus considered broad enough to ascertain the effect of increasing carrying capacity on wealth inequality and network size. Furthermore, the range of disaster frequencies we test (discussed below) is sufficient to allow the emergence of non-equilibrium dynamics at some settings. In our model, carrying capacity represents available biomass in the environment; more available biomass means larger numbers of animals can be sustained. For more information on our choice of carrying capacity range, and results from experiments beyond this range, see Section E in S1 File.

2) Herd growth rate:

We use a fixed growth rate of 10% per time step. In real life the growth rate of herds is modulated by the gestation period of animals, and the specific herd management/husbandry strategy adopted. It has been well established that herd size maximization is a common strategy in extensive pastoral nomadic systems, as a buffer against losses due to environmental disasters [23,28]. A rate of 10% seems high, but this is herd growth before reductions due to environmental disasters, and has an ethnographic basis. From 2013 to 2014, total livestock in Mongolia increased by 15%, and from 2014 to 2015 by 7.6% [27]. Therefore, in good years without widespread disasters, growth of around 10% is not unreasonable. In any case, experimentation showed that varying the growth rate did not produce new system dynamics. This is because what governs system dynamics related to growth rate is the ratio of average growth (the growth rate) and average loss (disaster frequency * disaster effect) that households face each time step. One set of dynamics held when average growth was greater than average loss, and another set held when average loss was equal to or greater than average growth. In this sense, varying the growth rate only varied the specific combination that would produce this threshold, providing no new information about system behaviour. This will be discussed further in the results section.

3) Disaster frequencies:

Households have between a 5% and 20% chance of suffering an individual environmental disaster per time step (and an equal chance of a system-wide disaster affecting all households simultaneously). Again, this broad range proved large enough to understand changes in system dynamics from low risk environments (5% frequency) to more high risk environments, and increasing disaster frequency beyond 20% did not result in any new noteworthy system behaviours. As extensive pastoral nomadic environments are characterized by risk, even under good conditions, the lowest risk frequency of 5%, or one disaster every twenty years, seems reasonable given available modern data (see [29,30]). Real world correlates to simulated environmental disasters include everything from droughts, snowstorms, freezing rain, disease, and human error or mismanagement.

4) Disaster intensity:

We use a fixed 50% reduction in herd size if a household suffers an environmental disaster. This may seem unrealistic, but as mentioned, the important dynamic between growth rates, disaster frequencies, and disaster effects was discovered to be average growth per time step (the growth rate), and average loss per time step (disaster frequency * disaster intensity). Therefore, the average losses per time step we test range from 2.5% (5% * 50%) to 10% (20% * 50%). Again, this range of average losses was sufficient to observe all noteworthy changes in system dynamics as the level of risk in the environment increases. To confirm this we also tested uniform random disaster intensities (0 to 100% reduction in herd size). Trends were identical but values were slightly fuzzier, as it became impossible to precisely calculate average loss per time step (now disaster frequency * a random number between 0 and 100).

5) Poverty threshold and patron threshold:

When a household’s herd size drops below 60 animals, they begin to look for a patron. Only households with herds larger than 800 animals can serve as patrons. Patrons transfer 60 of their own animals to a new client. If a patron’s own herd drops below 500, they lose their patron status and their clients. While real-world values will naturally vary depending on time, place, and context, these numbers have an ethnographic basis: Barth [31] locates 60 animals as the approximate poverty threshold among Basseri herders in present-day Iran, and similar values are located by researchers in Mongolia [22]. Likewise, in present-day Mongolia households with over 500 animals are comparatively rare, and regarded as wealthy. While the majority of Mongolian households currently own between 100 and 400 animals [22], more than half the population has left the herding economy (often through poverty) and urbanized. In previous lean times before mass urbanization, such as following the de-collectivization of herding in Mongolia after 1990, average herd size was under 60 animals per household [22]. It is important to note that in some sense our choices for these thresholds are unimportant; so long as they are held constant, the relative effect of independent variables such as carrying capacity and disaster frequency on network development can be measured.

#### Dependent variables

1) Wealth inequality:

In our simulation, wealth is measured by household herd size. While additional forms of wealth may have been employed in historical societies, our simulation aims only to understand baseline dynamics related to the herding economy. To measure wealth inequality we employ the gini index, a standard measurement of wealth inequality that varies from 0 to 1, with 1 representing perfect inequality. When the index value is 1 all animals in the system belong to only one household. A gini index of 0.8 roughly equates to 80% of total wealth in the hands of 20% of the households. The gini index is re-calculated at every time step, and its final value after 2,000 time steps is recorded. We measure wealth inequality using a more basic version of the simulation, in which households grow their herds and suffer environmental disasters, but do not interact with each other and form networks. This is because during interaction patrons redistribute wealth to their clients, masking total inequality.

2) Network size:

Every household who becomes a patron keeps a record of the clients that form its network. We measure patron-client network size by the number of nodes in each network. We record the largest number of clients (nodes) a single patron managed to maintain simultaneously each simulation run. The larger this number, the larger the network size that was able to form under the given parameter settings.

3) Network duration:

Besides recording the size of the largest network that developed during each run, we also record the number of consecutive time steps (out of 2,000) that it remained the largest within that run. This measures network duration or stability, and the average number of time steps the largest networks lasted over 100 runs at each set of environmental conditions is calculated and used for analysis.

## Results

**Fig 1:**
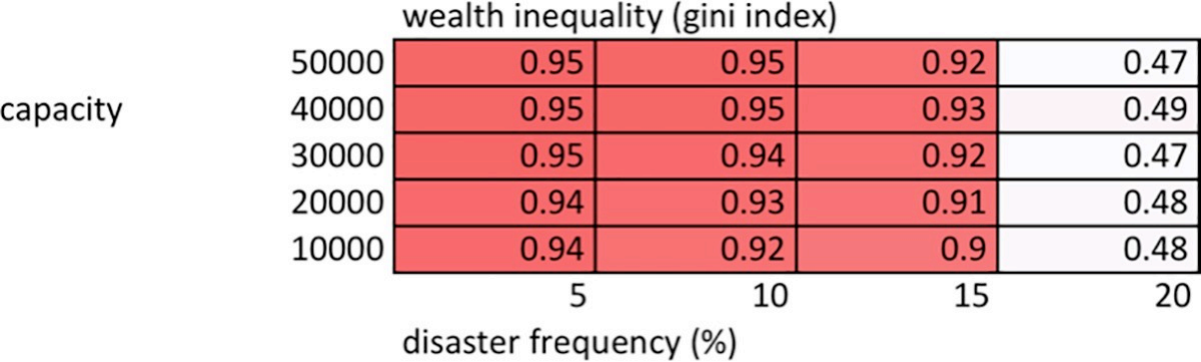
Wealth inequality (average values over 100 runs). Wealth inequality measured by gini index at the end of a run, averaged over 100 runs at each combination of carrying capacity and disaster frequency.

Fig 1 shows results for a version of the simulation that does not allow interaction between households and formation of patron-client networks, or household deaths. Households grow their herds every time step, which are then reduced whenever they suffer environmental disasters. It corresponds with steps 1 and 2 of the scheduling overview described above. Running only this portion of the simulation allows for a baseline analysis of the degree of wealth inequality that can develop, before households start to redistribute wealth through network formation. These results are average gini values from 100 simulation runs at each combination of carrying capacity and disaster frequency. Up to a 15% disaster frequency, wealth inequality is very high at all carrying capacities. At disaster frequencies of 20% and above, wealth inequality drops significantly. At a disaster frequency of 20%, average losses per time step are 10% (20% * 50%), equalling the growth rate. When average losses are equal to or greater than average gains, all households continually descend into poverty and are unable to distinguish themselves via wealth. Long term, these situations may be described as unsustainable. As discussed, examination of current empirical data indicates that disaster frequencies of between 5 and 15% are probably reasonable approximations of the extensive pastoral nomadic environment in the region. Within this range, our findings show that environmental heterogeneity engenders significant wealth inequality in the absence of social adaptations to mitigate it.

**Fig 2:**
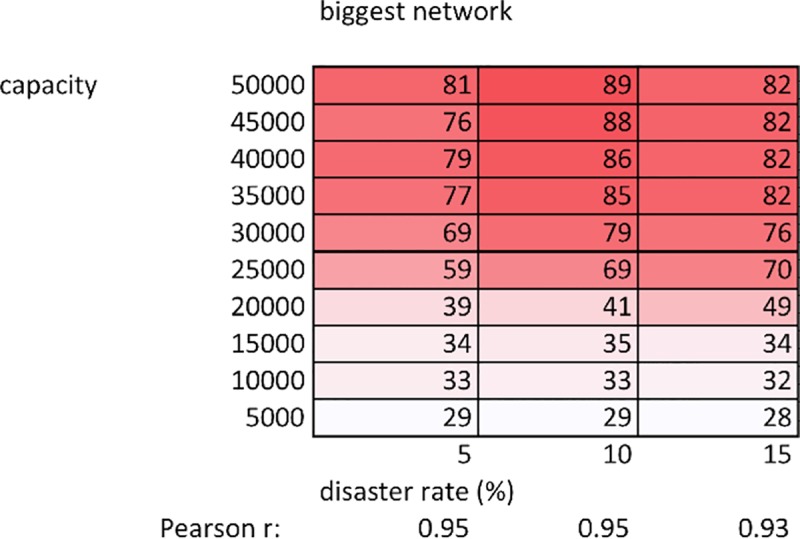
Network size (average values over 100 runs). Size of the largest network that developed during a simulation run, averaged over 100 runs, at each combination of carrying capacity and disaster frequency. Pearson r coefficients show the strength of relationship between network size and carrying capacity at each disaster frequency.

Fig 2 shows network size results for the full simulation. Between disaster frequencies of 5 and 15%, there is a strong correlation between carrying capacity and network size increase. As our ABM simulates 100 households, the largest possible network would feature 99 clients. The networks developing at the highest carrying capacities thus incorporate 80 to 90% of the households on the landscape. At disaster frequencies of 20% and above (when average losses meet or exceed average growth), households continually die off until the population is 0, preventing network formation and duration. For more details on this dynamic see Section F in S1 File.

**Fig 3:**
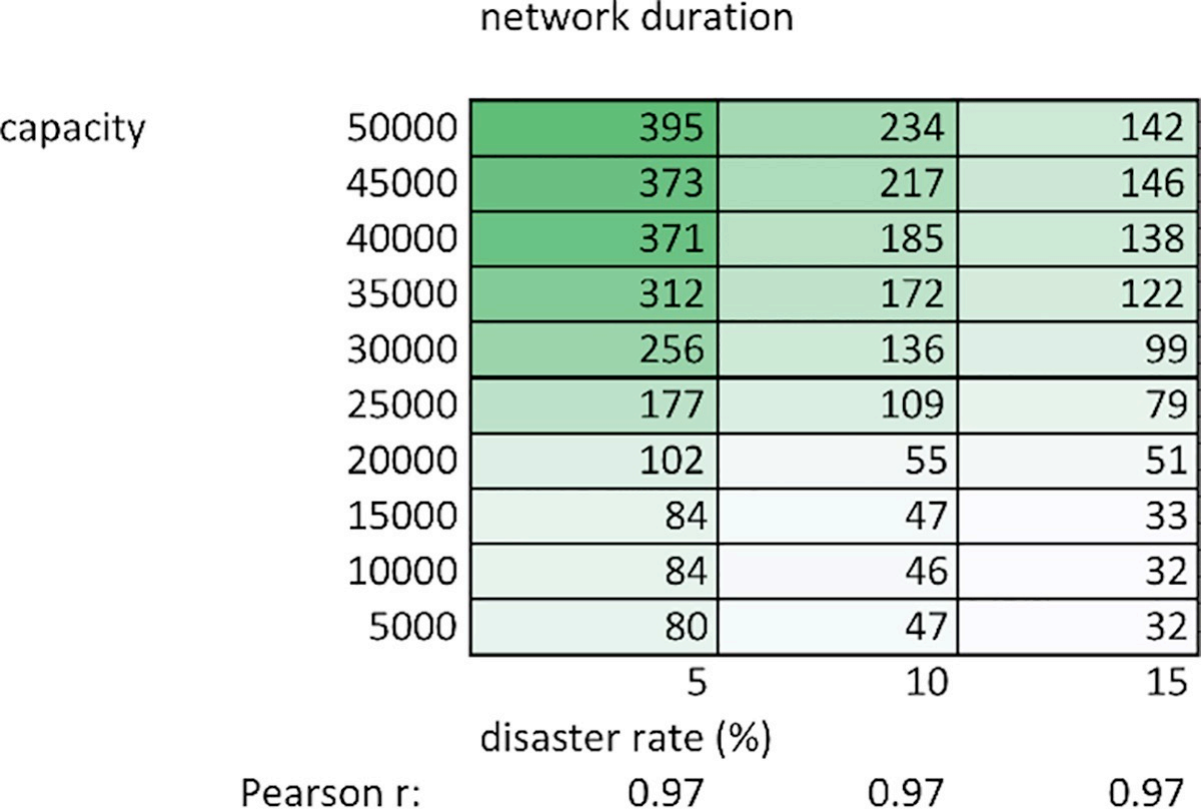
Network duration (average values over 100 runs). Duration in time steps of the largest network, averaged over 100 runs, at each combination of carrying capacity and disaster frequency. Pearson r coefficients show the strength of relationship between network duration and carrying capacity at each disaster frequency.

Fig 3 shows network duration in relation to carrying capacity, within the disaster frequency ‘sweet spot’ of 5 to 15%. Duration increases significantly with carrying capacity, and is highest at lower disaster frequencies. Therefore, as environmental conditions ameliorate, not only are networks getting significantly larger, but they are increasingly long lasting as well. However, even at the highest carrying capacities, wealthy patrons and their networks eventually always collapse, within the average time frame provided in Fig 3, to be eventually replaced by a new successful household. A time series analysis tracking patron wealth through individual simulation runs illustrates this rise-and-fall dynamic, and is presented in Section B in S1 File.

**Fig 4:**
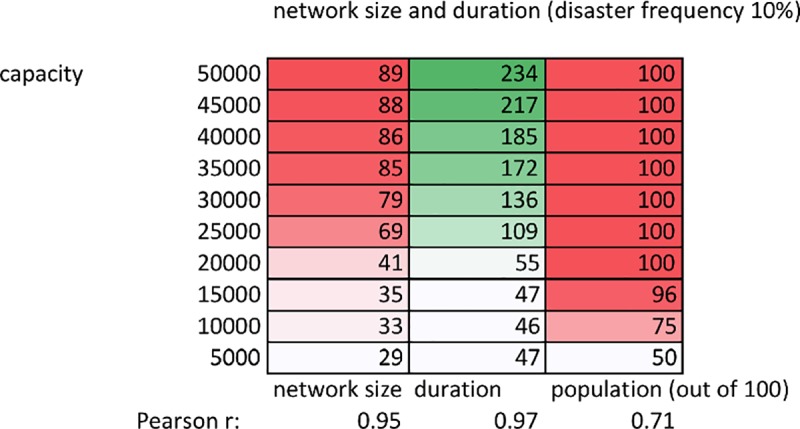
Network size and duration (average values over 100 runs). Network size, duration, and end population size (out of 100 starting households), averaged over 100 runs at each carrying capacity, with a fixed disaster frequency of 10%. Pearson r coefficients show the strength of relationship with carrying capacity for network size, duration, and population.

Fig 4 shows complete results at a disaster frequency of 10%. The right-hand column shows the remaining population at the end of a run, averaged from the results of 20 simulation runs at each carrying capacity. Households only died off at capacities of 15,000 and below. These results show that more household deaths at lower carrying capacities are not confounding the relationship between increasing carrying capacity and network size. At a capacity of 10,000, the largest network incorporated less than half of the surviving households, compared to the incorporation of more than 85% of households at capacities above 40,000. For more detailed analysis of death rates, including time series analyses of household deaths, see Section G in S1 File.

**Fig 5:**
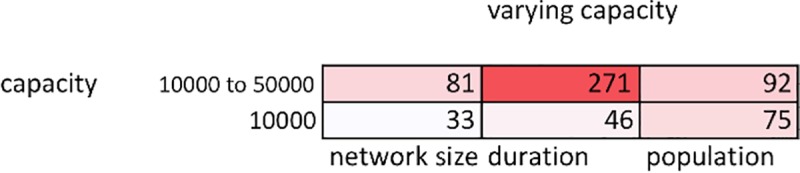
Network size and duration with capacity increase (average values over 20 runs). Network size, duration, and end population at a fixed carrying capacity of 10,000, and during an experiment raising carrying capacity to 50,000 if a large network developed. Averaged over 20 runs for each carrying capacity.

Fig 5 shows an experiment where whenever a network of 20 or more clients formed during runs at a capacity of 10,000, carrying capacity would be raised to 50,000 while that network remained in existence. We show average results from 20 runs of that experiment, again using a disaster frequency of 10%. Now a much larger and longer-lasting network formed in every run, and in 13 out of 20 runs this network incorporated more than 80 households. This experiment indicates that if available biomass increases during key moments when burgeoning networks are still small but beginning to form, the likelihood that a large network will develop is much increased.

### General sensitivity analysis with multiple regressions

To perform general sensitivity analysis on our ABM, we employ the commonly used method of multiple regressions [32], with results reported in Table 1. We perform three multiple regressions (one for each dependent variable) in which independent variables are carrying capacity (varied between 5,000 and 50,000) and disaster frequency (between 1 and 15%). The dependent variables are size of the largest network, duration of the largest network, and population size. To perform multiple regressions we use the SPSS statistics package, inputting 100 runs of results for each dependent variable at each disaster frequency, repeated at every carrying capacity. This gives 100 run results for every combination of carrying capacity and disaster frequency, totalling 4,000 runs. SPSS then calculates R-squared and R values, expressing the degree to which our two independent variables are correlated with each dependent variable. The Beta coefficients provide the direction of the relationship (positive or negative correlations). Figs 2–4 above also provide Pearson r coefficients for the relationship between each dependent variable and carrying capacity.

**Table 1:** Multiple regression results.

Dependent variable	R	R^2^	Capacity Beta	Disaster Beta
Network size	.773	.597	.665	.392
Duration	.703	.494	.411	-.570
Population	.699	.489	.639	-.284

The six *p* values for the *t*-tests of beta coefficients are highly significant (*p* < .001 in all cases).

These results show the dependent variables are highly sensitive to the employed variation in carrying capacity and disaster frequency. There is a strong positive relationship between carrying capacity and network size and duration, and a negative relationship between disaster frequency and network duration. Disaster frequency is positively correlated with network size, due to low network size at disaster frequencies of 1% (see Section F in S1 File). This result should be nuanced with the understanding that further increase in disaster frequency (to 20% and beyond), such that average losses per time step exceed average growth, results in no network formation, as all households die off. This is discussed in detail in Sections F and G in S1 File.

To expound further on model dynamics, we explain here how the independent variable of carrying capacity results in wealth inequality and changing network size and duration.

1) Wealth inequality

Development of wealth inequality requires 1) disasters that strike agents differentially, and 2) some level of carrying capacity. Without disasters, agents would grow their herds equally each time step, resulting in Gini indices of 0. As previously discussed, disasters must not be so high as to cause average losses to exceed average growth–in this case wealth inequality cannot develop, as agents cannot accumulate wealth. However, disasters alone are insufficient to result in high wealth inequality. Essentially, all agents would grow their herds on average each time step by the growth rate minus average losses (herd * (1.1 –(disaster frequency * 0.5))). Instituting any carrying capacity however, results in uniformly high wealth inequality (Fig 1). This is likely the result of two factors. Firstly, once carrying capacity is reached growth ceases, and thus the current level of inequality (due to stochastic timing of disasters affecting agents differentially) does not continue to average itself out over time. Secondly, while growth continues once disasters have reduced total herd size below carrying capacity, unless a wealthy agent’s large herd is struck by disaster, the overall losses are small, representing little opportunity for growth. Furthermore, while the order in which agents grow their herds each time step when total herd falls below capacity is random, once a wealthy agent grows their herd, remaining agents may not have the opportunity for growth, as capacity may already have been reached. This is due to the fact that 10% growth in a large herd represents in absolute terms a potentially larger number of animals than a 50% reduction in a smaller herd. In terms of fit with the empirical record, we feel this dynamic is realistic when dealing with a finite resource pool (common pasture with limited total biomass). In this context, wealthy agents have higher absolute growth potential (despite equal relative growth potential—e.g. 10%), and thus are able to monopolize limited opportunities for growth (limited by finite total available biomass).

2) Network size and duration

While wealth inequality does not vary substantially as a function of carrying capacity (Fig 1), network size and duration do (Figs 2–4). Since growth is relative (10% for all households, regardless of their absolute herd size), wealth inequality does not decrease as carrying capacity increases (Fig 1). While growth is relative, the lower patron threshold (500 animals) is absolute. This means that at higher carrying capacities, a wealthy patron requires a larger number of successive disasters before their herd decreases below this threshold and their network is dissolved. Furthermore, redistributing 60 animals to clients represents a significant burden on patron herd size, better absorbed by the proportionally larger patron herd at higher carrying capacities. For example, a patron with 89 clients will, at a minimum, have distributed 60 animals to each client once, representing 5,340 animals. While agent herds will more frequently dip below the poverty threshold (60 animals) at lower carrying capacities (as wealth distribution is the same as at higher capacities, but total system animals are fewer), this is more than counterbalanced by the lower probability that a patron is able to support so many clients without collapsing. At higher capacities, not only do patrons have a larger buffer before collapse, but also their clients will require less redistribution to maintain. For example, when a patron possesses 80% of total system wealth, the remaining 20% at higher carrying capacities will be greater than the sum of poverty threshold herd sizes amongst the remaining agents. In other words, at the highest carrying capacities, after initial redistribution of animals to a new client, a patron is less likely to have to support that client again. Combining the above factors increases the probability that, at lower carrying capacities, a patron’s network will be smaller, and will dissolve more rapidly (Figs 2–4).

## Discussion

We compare model results with the Mongol (1206–1368 CE) and Xiongnu (209 BCE– 48 CE) empires. We also discuss common confounding variables that may act upon our simulation dynamics in empirical cases, and how our type of simulation can be applied to empirical analysis. We stress that by examining multiple case studies we are not implying a necessary or exclusive correlation between positive climatic change and nomadic empire creation. This latter is a multivariate process, and the effects of climate may be confounded or outweighed by any number of political, social, and cultural variables emerging from a long causal chain of historical particulars. Nonetheless, our approach is to use simple, abstract simulations involving as few variables as possible. This builds up an understanding of relationships that allows for a better appreciation of the ultimate effect of additional variables. Additionally, the more abstracted a model, the more potential it has to be applied and analyzed in a variety of particularistic scenarios.

In applying our model, we are primarily interested in cases where empirical evidence has already suggested enhanced environmental productivity did correlate with documented moments of nomadic empire creation, hierarchy, and centralization. Comparing model processes with empirical data is a great challenge, since the archaeological and historical record on the particulars of the emergence of these empires is so scarce. In the case of the creation of the Mongol Empire, Di Cosmo [33] notes that the only early historical source, the *Secret History of the Mongols*, is of unknown reliability. This is compounded by its overwhelming focus on lauding the military exploits of Genghis Khan and his allies, at the expense of elucidating critical background factors involved in the economics and logistics of network creation and maintenance. In cases such as these, we feel simulation is an ideal tool to experiment with mechanisms that can connect the dots between a) an empirically observed environmental context, and b) an empirically observed socio-political phenomenon. Two of these correlations between climate and empire (the Mongol and Xiongnu periods) happen to be the two largest and longest lasting nomadic empires in the historical record. As such, they are our primary case studies. We are also interested in evidence for the adoption of strategies designed to mitigate the effect of climatic downturns on hierarchical networks and polities among nomads, and cases where the effects of climate have been confounded by other political, social, or cultural variables.

### Mongol Empire (1206–1368 CE)

According to recent dendroclimatology research, Mongolia experienced drought from 1180 to 1190 CE, and an unprecedented pluvial from 1211 to 1225 CE, along with above-average temperatures [5]. The drought corresponds with historical evidence for violent tribal warfare within Mongol society, and the warmer and wetter period corresponds with Genghis Khan’s early expansion and consolidation of the Mongol Empire. Pederson et al. [5] hypothesize the pluvial would have aided expansion due to lusher pastures easing the logistics of military conquest. They also propose that the breakdown of social order prior to Genghis Khan’s rise to power would have been exacerbated by drought, clearing the way for a new polity to emerge. Putnam et al. [6] adopt a more macro perspective on climate reconstruction, suggesting that little ice age cooling led to glacier expansion across the Eurasian continent from, at the latest, 1180 CE through to nineteenth century. In Mongolia, they argue this led to a southward push of snow lines, grasslands, and concomitant wetting of Inner Asian deserts, substantially increasing available biomass.

**Fig 6:**
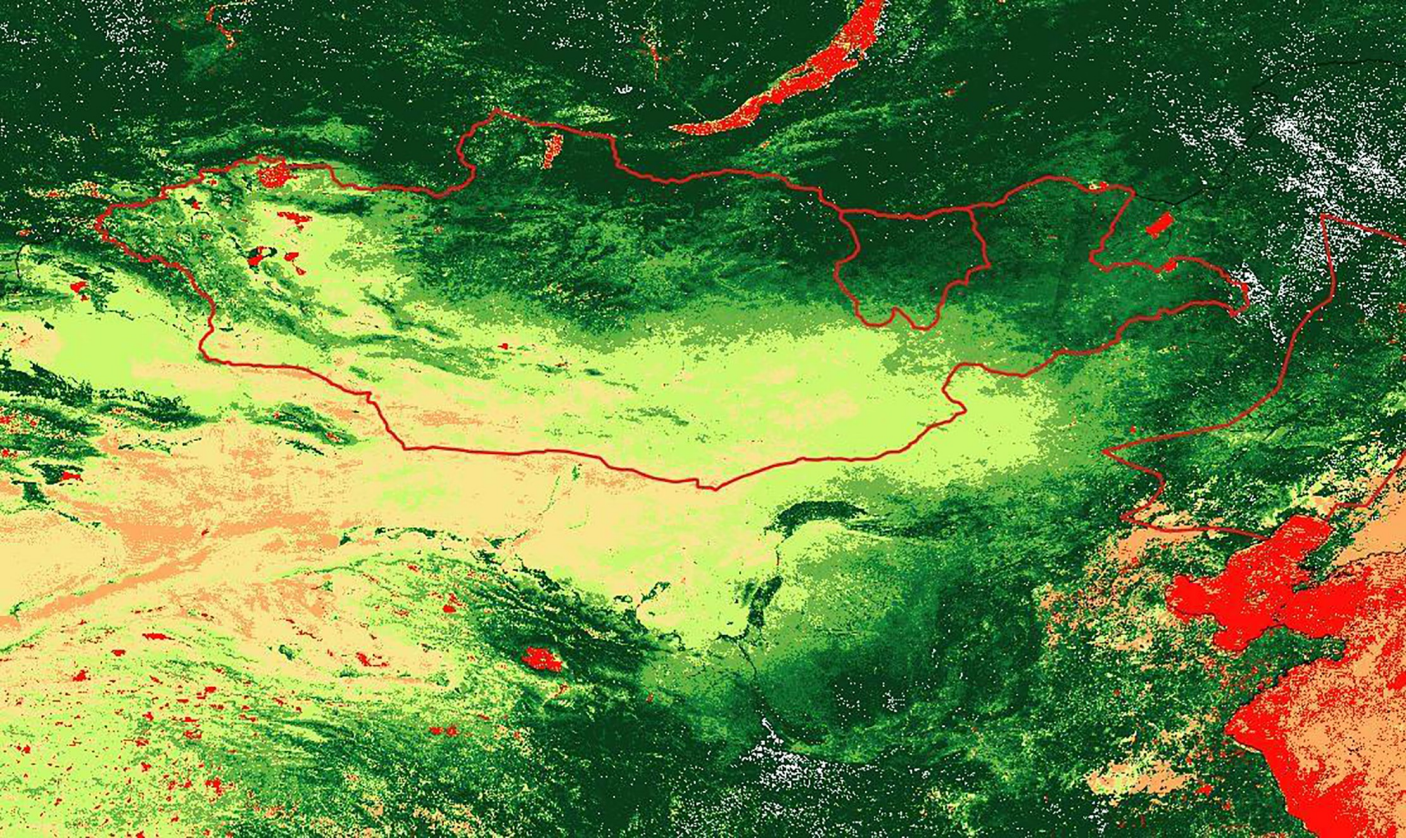
NDVI data, Mongolia. Sample NDVI satellite imagery for July 2014, with Mongolia and Khentii province outlined in red. Darker green indicates higher biomass. Our analysis of imagery suggests Khentii generally features NDVI values 75% higher than the Mongolian national average.

Climate data suggesting a warmer and wetter period in the early years of the Mongol Empire fits well with our model predictions, as these are the conditions characterized by increased rangeland productivity that increase the probability of large stable network formation (Fig 4). Furthermore, the heartland of the early Mongol Empire was between the Kherulen and Onon rivers in Mongolia’s present-day Khentii province, home territory to Genghis Khan’s Khamag Mongol confederation. In the present, Khentii is known as one of the most productive regions in Mongolia; in 2016 it had 789 households with over 1,000 head of livestock, placing it second in Mongolia, at double the national average [27]. The relatively high carrying capacity of Khentii pastures is borne out by NDVI satellite imagery, reflecting photosynthesis capacity per unit land, shown in Fig 6.

While we experiment with poor climatic conditions followed by positive climate change and enhanced productivity (Fig 5), the preceding period of negative productivity (drought) observed by Pederson et al. [5] is not a necessary component of large network formation in our model. Even in simulations run exclusively in high productivity, low risk environments, network duration shows regular turnover in patrons, such that roughly ten different large networks could be expected per 2,000 time cycles under these parameters (Fig 3). In the specific case of the Mongol Empire, however, a chaotic breakdown and civil violence (which we do not model), potentially exacerbated by drought, could undoubtedly have played a role in Genghis Khan’s emergence as a new leader.

The actual process simulated in our model, of differential success of herding agents followed by redistribution of animals from rich to poor households, is extremely difficult to identify in historical sources, and particularly in archaeological data. The aforementioned *Secret History of the Mongols* (SHM) may be an embellishment of military valour, but social and cultural references would probably have been recognizable to contemporary readers, and provide indirect evidence for processes seen in simulation. Chapters covering the reign of Ogedei Khan (1229–1241 CE) make passing reference to Ogedei mandating the collection and redistribution of sheep from aristocratic and military leaders to poorer households (SHM 279–280). Aristocratic characters in the *Secret History* also make occasional references to the camp locations and movements of specialist herders of horse, sheep, and other animals working for them, implying contractual herding agreements (e.g. SHM 118, 152, 169). Wright [34] has recently argued that the characteristic empty walled enclosures of the Mongol Empire period functioned as animal collection centres, in an early example of nomadic pastoral taxation and surplus. Regular taxation of livestock at these enclosures during seasonal migratory movements indicates one way a centralized power, or patron, could redistribute wealth where it was needed to maintain followers and authority. While the *Secret History of the Mongols* is equivocal regarding exactly how Genghis Khan provisioned and maintained his growing network of followers [33], Pederson et al. [5] note that increased carrying capacity in central valleys would have permitted larger, more permanent habitation sites. This matches with historical and archaeological evidence for *ordos* (large camps/territories) of elite leaders, along with other permanent structures, during the Mongol Empire period (SHM 96, 104; Rubruck XII; [34,35]). Taken in isolation, this sparse data would be difficult to form into a coherent theory of empire creation, but coupled with simulation experiments, a pattern of network formation based on redistribution of unequal herding surplus seems plausible. This is further supported by data from the ethnographic era on the development of patron-client contract herding relations in a variety of nomadic pastoral societies [9,31].

Other elements of the Mongol Empire and its rise provide an opportunity to understand the complex interplay between the simple relationship our model demonstrates, and its interaction with socio-political variables. While a climatic upturn at the end of the twelfth century increased the probability of a nomadic polity, political occurrences external to the Mongolian herding landscape were also critical. From the tenth to twelfth centuries, Mongolia was a territory within the Khitan Liao Empire, a status severely curtailing any indigenous polity creation. The collapse of the Liao after military defeat at the hands of the Jurchen Jin Empire subsequently created a relative power vacuum on the Mongolian steppe, which grew larger following Jin withdrawal in the mid twelfth century [36]. External political and military events played a critical role in creating an environment of relative freedom from confounding external variables, in which network dynamics among herders could play out against the backdrop of an enhanced climate.

Likewise, socio-political structures, whether by design or by accident, may sometimes function to mitigate effects that emerge in our model, such as the rapid breakdown of large networks during climatic downturns. A noteworthy feature of the later Mongol Empire, in comparison to earlier nomadic polities, was its adaption of sedentary features such as fixed urban centres. These included the capital city of Karakorum, and the later capital of Dadu (present-day Beijing). These cities could be supplied with and store grain from conquered Chinese agricultural areas [37]. Official documentation from the Mongol Yuan Dynasty makes repeated mention of large numbers of Mongolian herders, made destitute by environmental disasters such as winter snow storms, entering these centres and being granted grain or cash [38,39]. An adaptation of our present simulation model includes externally provisioned urban centres that poor agents can migrate to instead of seeking herding patrons [40]. Results indicate these centres substantially increase the resilience and duration of remaining herding networks during short-term climatic downturns, by providing a runoff zone for agents that would otherwise drain the energy resources of their patrons. Mongol Empire urban centres are a useful example of how an additional variable may confound the relationship between climate and networks expressed in our model. However, understanding of the effect of this new variable is substantially enhanced by prior knowledge of the simple relationship between climate and networks we report here.

Given that our results indicate relative probabilities of large network creation, it does not surprise us to find that the largest of all nomadic empires 1) originated in a part of the pastoral world that is relatively lush [25], 2) centered on a sub region of Mongolia that is arguably its most productive, and 3) arose during a time period with an uncharacteristically favourable climate for herding. While these correlations may seem fairly obvious in hindsight, this is not the case, given longstanding negative climate theories of nomadic empire creation. The history of this intellectual tradition stretches back at least to thirteenth century historian Ibn-Khaldun’s *Muqadimmah*, where he identified the relative privation of nomads as the driving force behind nomadic conquest and state formation. This has been coupled with the longstanding viewpoint of sedentary societies concerning the relatively primitive character of nomadic social organization and its dependency on sedentary neighbours for surplus energy.

### Xiongnu Empire (209 BCE– 48 CE)

The Xiongnu Empire is the first historically documented empire created by nomads in present-day Mongolian territory, and the longest lasting, presenting a useful comparison with the later Mongol period. Ge, Hao, Zheng, & Shao [41] use proxies including sediment, tree rings, ice cores, and historical documents to reconstruct regional temperatures on a decadal timescale over a 2000-year period. Similar to the initial years of the Mongol Empire, they find that there was a significant warm period from roughly 200 BCE to 180 CE. Likewise, precipitation research indicates this warm period was also wet [42,43]. More specific to the Xiongnu, Houle [44] has collected relevant environmental, archaeological, and faunal data from multiple regions of Mongolia to test the Mongol Empire model of climate and empire creation developed by Pederson et al. [5] on the Xiongnu period. He finds that lake core data from the central Mongolia/Khanuy Valley area indicate warmer and wetter conditions during initial creation and expansion of the Xiongnu polity. Faunal analysis in the Khanuy Valley indicates this led to expanded rangeland productivity, with an estimated threefold increase in the large domestic mammal population, and a doubling of the human population during the initial Xiongnu period. This region is associated archaeologically with elite Xiongnu burials and is generally taken as the heartland of the Xiongnu polity. In contrast to the Mongol Empire climate model developed by Pederson et al. [5], Houle finds a) no evidence of poor environmental conditions prior to the initial Xiongnu period, b) no evidence of violence preceding Xiongnu polity formation, and c) no evidence for clear centralization of power in a manner comparable to the Mongol Empire.

We agree with Houle’s conclusion that climate cannot be thought of as the sole driver of empire creation in the Xiongnu case [44]. Each empire is a multivariate phenomenon with different particulars governing emergence. Even in our model, which tests the effect of environmental productivity on network formation, climate change is not the sole driver, as we observe the formation of large networks (albeit at differing rates of probability) under a wide variety of environmental parameters. Rather, wealth inequality, or more precisely the ability of some agents to gather, store, and redistribute surplus energy, is ultimately the driver of hierarchical network formation in our model. What our simulation shows is that a rise in environmental productivity increases the probability of large network formation. In this context, our findings provide interesting insights into the Xiongnu, who were able to maintain an uncharacteristically large and long lasting nomadic polity during a period of documented warmth, wetness, and increased rangeland productivity.

As Houle [44] notes, archaeological evidence on the degree of centralization in the Xiongnu polity is unclear in comparison to the Mongols, whose politics have greater historical documentation, and whose material remains include capital cities. However, nomadic empires before the adaption of sedentary features such as fixed capitals may present little to no material evidence of centralization or hierarchy for future archaeologists. For example, our virtual simulation space features redistribution of herds from wealthy patrons to poorer clients, forming hierarchical networks. If this simple space with no other additions were an archaeological landscape, it would present the appearance of a dispersed homogeneous population of fairly egalitarian herding households. Archaeological evidence aside, historical primary sources, while providing no information on the initial formation process of the Xiongnu Empire, certainly document its hierarchical, centralized network of power. This is represented by the military decimal system, consisting of leaders and groups of 10, 100, 1,000, and 10,000, with the *Shanyu*, or emperor, at the top (*Shiji* 110). There is longstanding debate concerning the reliability of primary sources discussing Xiongnu society, due to the ethnic Chinese origins of these texts [45]. In this case however, the description of the Xiongnu decimal system is identical to the Mongol Empire decimal system over a thousand years later, which is more reliably documented in indigenous Mongol, European, and Persian primary sources (SHM 224; Juvayni I 23; Carpini V 22, VI 2; Rubruck IV, XII). The decimal system thus appears to be a long lasting steppe tradition and a reliable observation from Xiongnu primary sources. As the non-military, administrative role of the decimal system for the Mongol period has been well argued [35,46], it is likely an elegant mathematical representation of a hierarchical system of livestock herding in use during the Xiongnu period.

However, we are agnostic regarding degrees of centralization amongst the Xiongnu elite relative to the Mongol Khans and Emperors, so long as there were elites with networks. Historical documentation and tomb burials strongly suggest this to be the case. Our abstracted simulation could be considered analogous to the development of a single regional network. We do not simulate horizontal interactions between multiple pre-existing hierarchical networks, which may operate under a variety of different dynamics, and represents an area for future research. Houle’s [44] conclusion based on archaeological findings that the emergence of the Xiongnu polity, unlike the Mongols, was not characterized by violence, fits well with our model, which simulates only non-violent economic exchanges between agents. Honeychurch [47,48] characterizes the lack of violence preceding the emergence of the Xiongnu polity as indicative of the long-term peaceful agglomeration of networks. Makarewicz’ [14] pioneering zooarchaeology research in Mongolia also finds strong evidence in favour of nomads being able to develop highly mobile surplus energy (in the form of herd animals) during the Xiongnu period. This ability to accumulate and redistribute energy is of course critical to the functioning of our network creation model, and supports arguments for the intrinsic capability of nomadic pastoralists to develop social complexity.

Beyond the Xiongnu and Mongol Empires, numerous pastoral ethnic groups in the region have coalesced into polities of varying sizes and are documented with varying degrees of reliability in Chinese written sources. Particular area and chronological specialists will be needed for future research to weigh different causal factors, including climate, in the emergence of these groups. Thus far, a small number of large-scale quantitative projects have attempted to correlate environmental data with nomadic political activity over long time spans. Su et al. [7], make use of multiple climate reconstructions (namely [41–43,49,50]), and find that periods of warfare between East Asian nomads and neighbouring sedentary societies occurred most frequently during climatic upturns, in contrast to previous models proposing nomadic warfare as a reaction to resource scarcity [51]. According to Su et al. [7], this divergence is due to their focus on warfare between nomadic and sedentary groups, and not violence within groups. In other words, a unified nomadic society warring against a foreign power occurs more frequently in climatic periods of enhanced productivity. The primary explanatory mechanism proposed is that improved pastoral productivity in a favourable climate provided a foundation of strength and population growth to nomadic polities [8]. While we do not simulate warfare, our results support this proposal for the enhanced backdrop to successful warfare and social cohesion that increased environmental productivity could provide. As in our simulation model, this study is expressed in terms of probabilities. A climatic upturn makes warfare between a unified nomadic group and sedentary neighbours more likely, with statistical significance, but warfare between groups can and does occur across the full climate spectrum.

As previously stated, we do not expect that the examination of further case studies will continuously result in findings supporting our model predictions. Not only do our results include the formation of large networks under a wide variety of conditions, but multiple additional variables we do not simulate are also involved in real world polity creation. In this regard, traditional theories predicting climatic downturns would correlate with the creation of nomadic empires have theoretical merit (and probably some empirical validity, given a large enough sample of cases). In our model, very poor environmental conditions (where average losses per time step are equal to or greater than average herd growth) do not result in large network formation because no agents are wealthy enough to maintain networks, which quickly collapse. In this environment, additional variables such as military and political organization and charismatic leadership could potentially counterbalance this outcome. Subsequently, extraction of surplus energy by force from neighbouring societies could be used to maintain a social hierarchy [11].

Since our model supports network creation mechanisms under a variety of conditions, increased rangeland productivity is not the actual mechanism of network creation; rather, it increases the probability of creation. The actual mechanism is environmental heterogeneity and the subsequent unequal accumulation of surplus energy, coupled with its storage and redistribution in a highly mobile form (herd animals). As such, our model provides a mechanism for nomadic social complexity and polity formation even in the absence of positive climate events. This finding is supported by recent research recognizing the critical influence of animal domestication and the development of pastoralism in the rise of wealth inequality in Eurasia [52].

## Conclusion

Many different paths to nomadic empire or polity formation have undoubtedly occurred, and this paper makes no claims that ameliorating environmental conditions are a prerequisite. In our simulation, substantial networks (incorporating more than 40% of surviving households) formed even under relatively poor environmental conditions. Inter-group violence and other political tactics are not simulated, and could lead to polities or empires under varying sets of preconditions. Our results are particularly pertinent to situations where empirical evidence indicates substantial expansion of available biomass during or prior to nomadic polity expansion, or to polities that originated out of relatively favourable sub-regions. Under these conditions, we propose an explanatory mechanism for this correlation, whereby an increase in available biomass and heightened wealth inequality increase the probability of larger and longer lasting hierarchical livestock herding networks. This can occur regardless of whether preceding conditions were poor or favourable, and in the absence of large-scale violence or social upheaval. More generally, we show how a variety of heterogeneous environments can result in the unequal accumulation of surplus energy and the subsequent emergence of social hierarchy. These dynamics are relevant for the initial creation of polities and empires in livestock herding societies. They add to recent research on the internal capacity of nomadic society for social complexity, and on the effects of increased biomass on the expansion of nomadic empires.

## Supporting information

S1 File Supporting information. (DOCX)

S2 File NetLogo code. (DOCX)

S1 Video Sample simulation run visualization. (MP4)
