# Supplementary material for: Modeling environmental variability and network formation among pastoral nomadic households: Implications for the rise of the Mongol Empire
Source: PLoS One. 2019 Oct 10;14(10):e0223677. doi: 10.1371/journal.pone.0223677 (PMC6786613; doi:10.1371/journal.pone.0223677)
Supplement: S1 File — (DOCX) [file pone.0223677.s001.docx]

**Supporting Information S1 File**

This file contains information on:

a) Coefficient of variation for minimum run validation

b) Herd size time series of top patrons

c) Average household metrics

d) Experimentation with number of time steps

e) Experimentation with carrying capacity range

f) Experimentation with disaster frequency range

g) Population size time series analysis

h) Simulation animation explanation

**a) Coefficient of variation for minimum run validation**

To determine the minimum number of simulation runs required to capture variance at each parameter combination, we use the coefficient of variation method (Lee et al., 2015; Lorscheid et al., 2012). This coefficient (cV) is simply the ratio of the standard deviation of a sample (σ) to the mean (μ) of that sample (cV = σ / μ). This coefficient is calculated and compared for increasing numbers of runs, until it stops varying beyond a fixed *epsilon* (*E*), or threshold. In our case, *E* is set to 0.01. Using this method showed that 100 runs at each parameter combination was sufficient to capture variation. A sample of these calculations is provided below for the dependent variable of network duration at a carrying capacity of 50,000 and a disaster frequency of 10%:


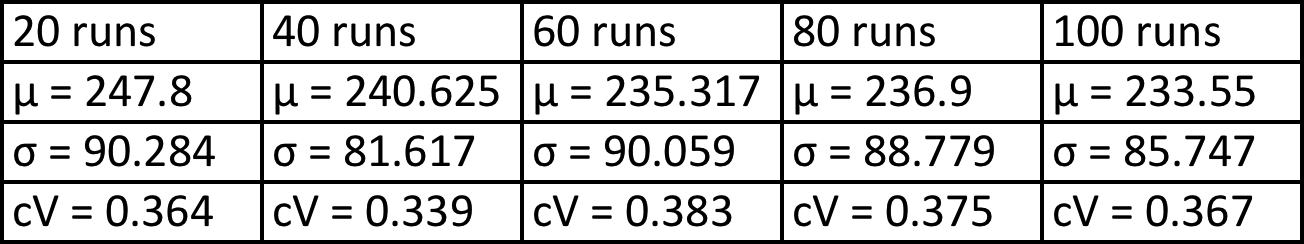


The difference between the cV at 100 and 80 runs is < 0.01 (0.008), as is the difference between the cV at 80 and 60 runs. This indicates that at this parameter combination, 60 runs would have been sufficient. However, this calculation must be performed for each parameter combination and the minimum number of runs should be selected using the parameter combination requiring the highest minimum number. In our case this proved to be 100.

**b) Herd size time series of top patrons**

Figs A-I show time series plots of herd size from individual 2,000 time step runs for the top three patrons in each run. Quantifying ‘top’ patrons can be done in multiple ways (e.g. most number of time steps as the wealthiest household, or most number of time steps as patron of the largest network). Generally, the same households are in the top three for most such metrics (see figures J-L). For figures A-I we use the number of time steps spent as patron of the largest network in the system (including non-consecutive time steps). Coupled to each figure is a time series plot of the largest network at each time step during the same run, showing, as expected, that the largest networks correlate temporally with the wealth (herd size) of the patron of the given network. These figures show that patrons rise and fall with regularity, and the largest network can develop at any time throughout the run. Even at high carrying capacities, it is a certainty that even the largest networks and wealthiest patrons will eventually collapse, within the number of time steps shown in main text Fig 3 (maximum network duration by carrying capacity).

**Fig A: 50,000 carrying capacity – 10% disaster frequency – Run 1**


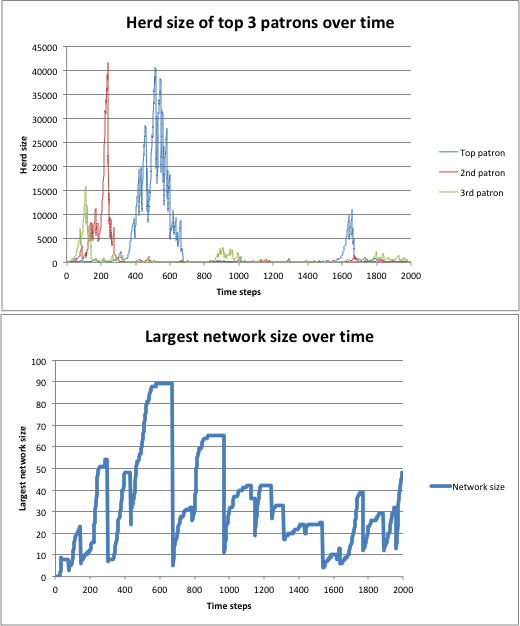


**Fig B: 50,000 carrying capacity – 10% disaster frequency – Run 2**


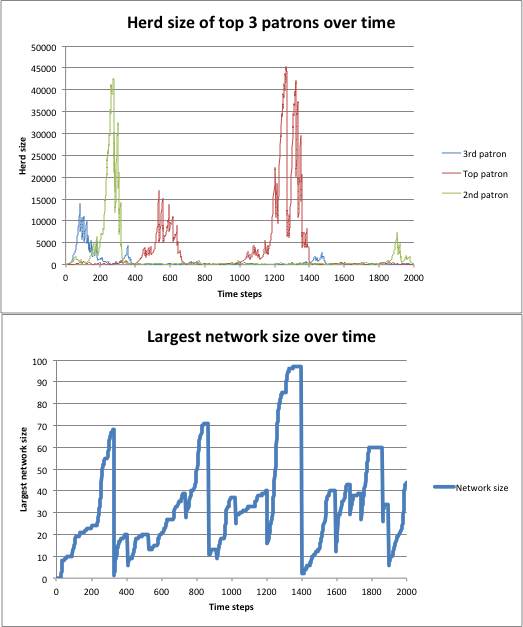


**Fig C: 50,000 carrying capacity – 10% disaster frequency – Run 3**


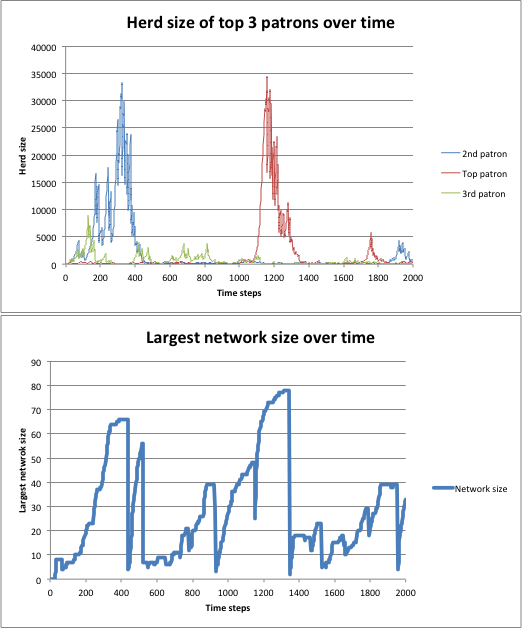


**Fig D: 30,000 carrying capacity – 10% disaster frequency – Run 1**


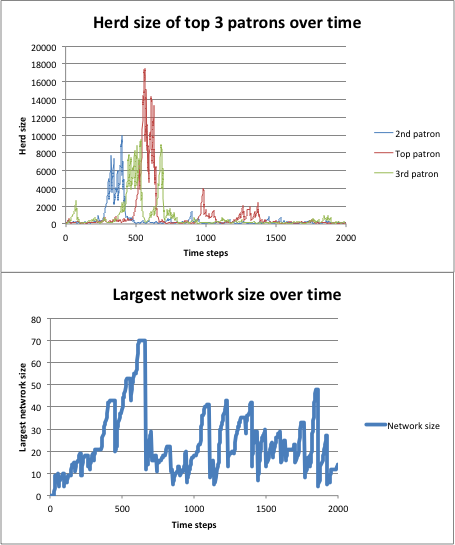


**Figu E: 30,000 carrying capacity – 10% disaster frequency – Run 2**


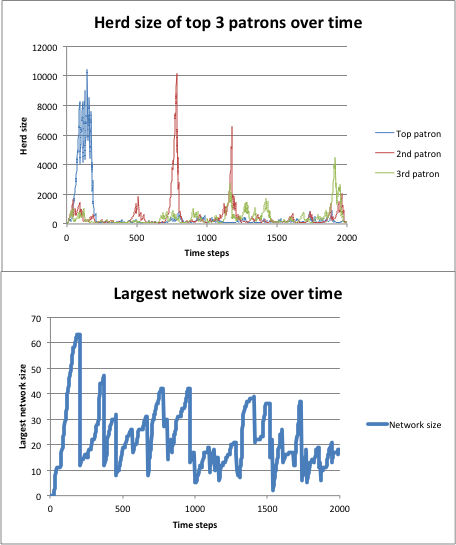


**Fig F: 30,000 carrying capacity – 10% disaster frequency – Run 3**


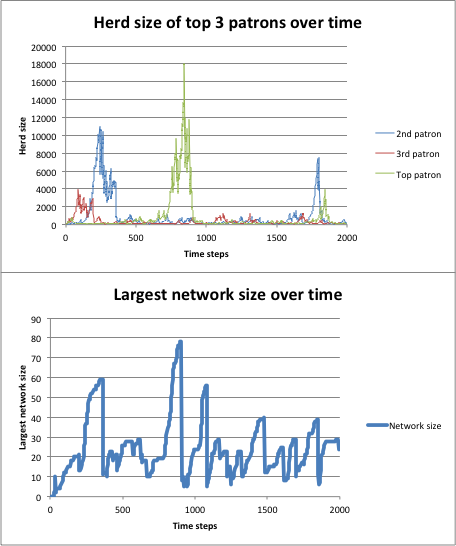


**Fig G: 10,000 carrying capacity – 10% disaster frequency – Run 1**


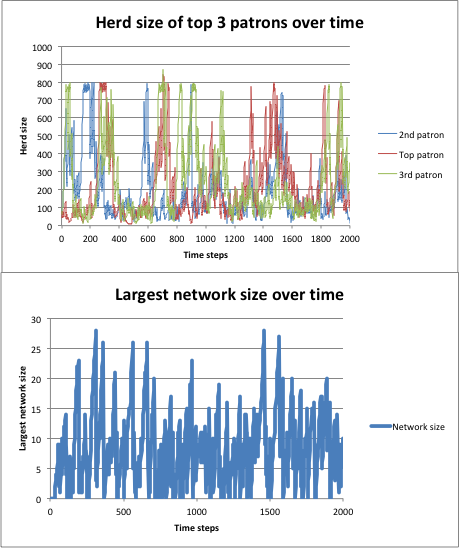


**Fig H: 10,000 carrying capacity – 10% disaster frequency – Run 2**


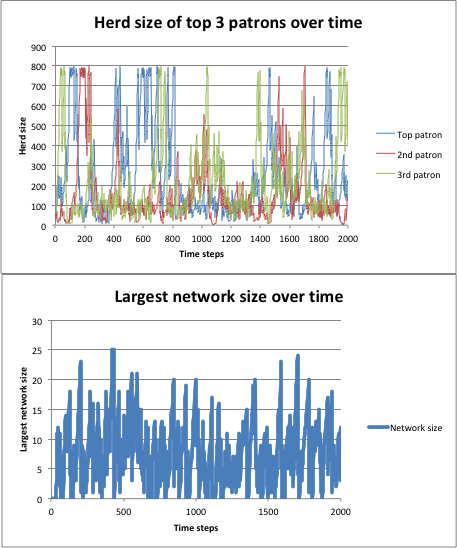


**Fig I: 10,000 carrying capacity – 10% disaster frequency – Run 3**


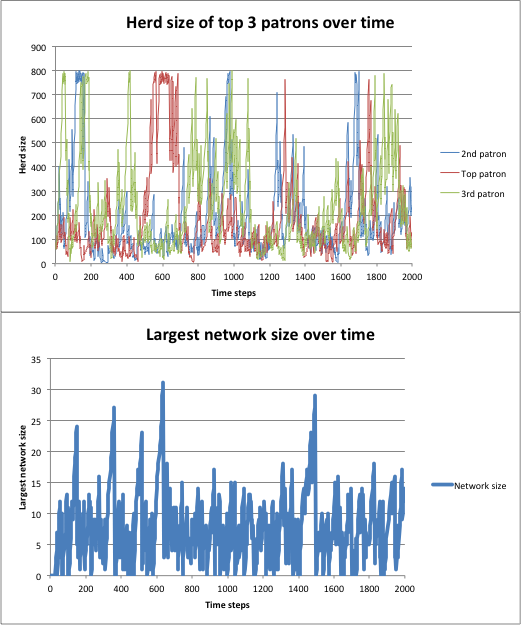


**c) Average household metrics**

During each run, households keep track of 1) how many time steps they spent as the wealthiest household in the system, 2) how many time steps they spent as patron of (at that time step) the biggest network in the system (including non-consecutive time steps, unlike our network duration metric), and 3) how many time steps they spent as patrons (regardless of their network size). All 100 households are ranked according to these metrics at the end of each run. We then average the value at each of the 100 ranks over 100 runs. This gives, for example, the longest average number of time steps a household spent as a patron, second longest, third, etc. Figs J-L show these results at carrying capacities of 50,000, 30,000, and 10,000 for each metric. Each metric shows that, at all carrying capacities, multiple households rise and fall throughout the 2,000 time steps. As carrying capacity rises, a smaller number of households gain increasing advantage, with the most dominant household achieving significantly higher time steps for each metric. These results are further expounded in main text Fig 3 and by using multiple regressions for general sensitivity analysis.

**Fig J: Households ranked by average time steps as wealthiest (10% disaster frequency)**


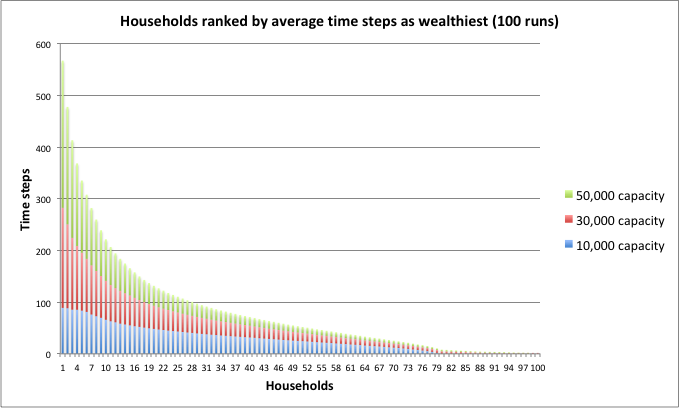


**Fig K: Households ranked by average time steps spent as patron of largest network (10% disaster frequency)**


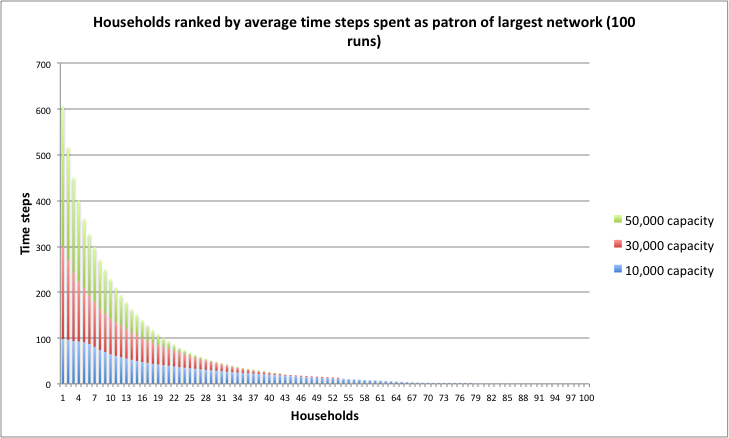


**Fig L: Households ranked by average time steps spent as patron (10% disaster frequency)**


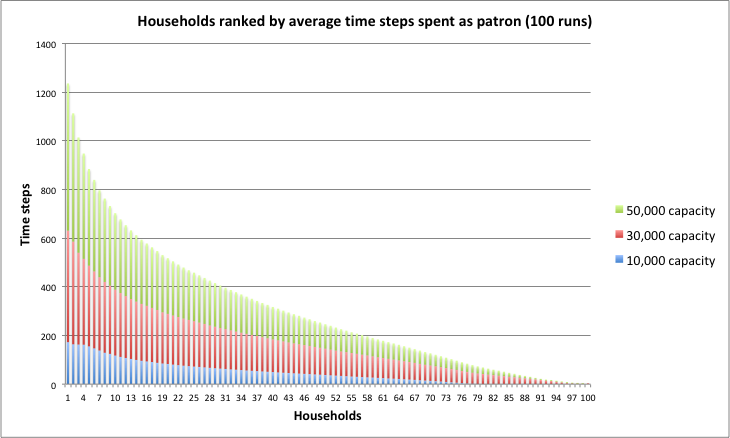


**d) Experimentation with number of time steps**

In our simulation we conceive of time steps as roughly equivalent to years, as this is how we derive our growth and disaster rate probabilities from ethnographic evidence. Because of this, we choose 2,000 time steps, as a) it proved a long enough period to allow several large networks to rise and fall at each carrying capacity (see Figs A-I), and b) roughly coincides with the time span Inner Asian nomadic societies and empires are historically recorded as a major political force (~300 BCE – 1700 CE). Nevertheless, we experiment with a significantly higher number of time steps (10,000), to ensure we are not missing any important dynamics that take longer to develop. Fig S shows average values (from 100 runs of 10,000 time steps) for network size and duration through our carrying capacity range at a 10% disaster frequency. In comparison to main text Fig 4, values are slightly higher (more time steps increase the chance of low probability occurrences), but the trend with increasing carrying capacity is identical. Fig N is also comparable to Figs A-C, indicating that the largest, longest lasting networks can appear at any time during a run. Because 10,000 time steps does not alter the trend, we use 2,000 time steps for our main analyses, given 1) it is more empirically realistic, and 2) it saves considerable time and computational power relative to 10,000 time step runs.

**Fig M:**


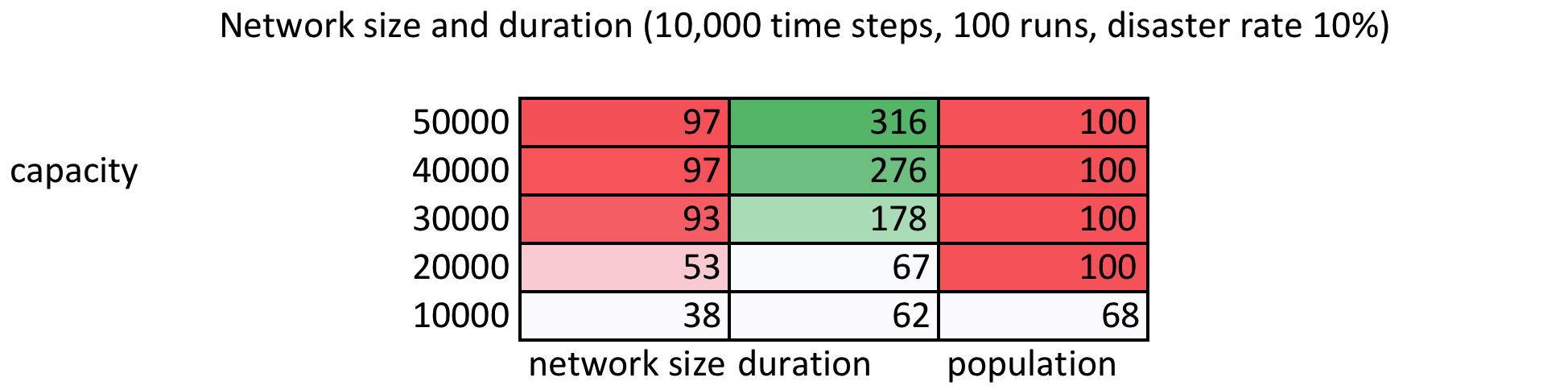


**Fig N: Sample run of 10,000 time steps (50,000 carrying capacity, 10% disaster frequency)**


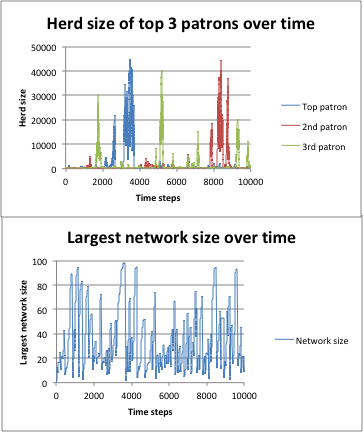


**e) Experimentation with carrying capacity range**

The upper limit of our carrying capacity range (50,000 animals) is also derived ethnographically, relative to the number of households (100) that we simulate (see main text pages 9 and 10). However, here we report results with increased carrying capacities (up to 200,000 animals) to ensure we are not missing interesting dynamics. Fig O shows that the trend seen in our main results for network size and duration continue as carrying capacity increases. Curve fitting reveals that, within these parameter ranges, both relationships correspond to a polynomial function (Fig P). We perform our main analyses using a 50,000 animal upper limit, as it is empirically defensible.

**Fig O:**


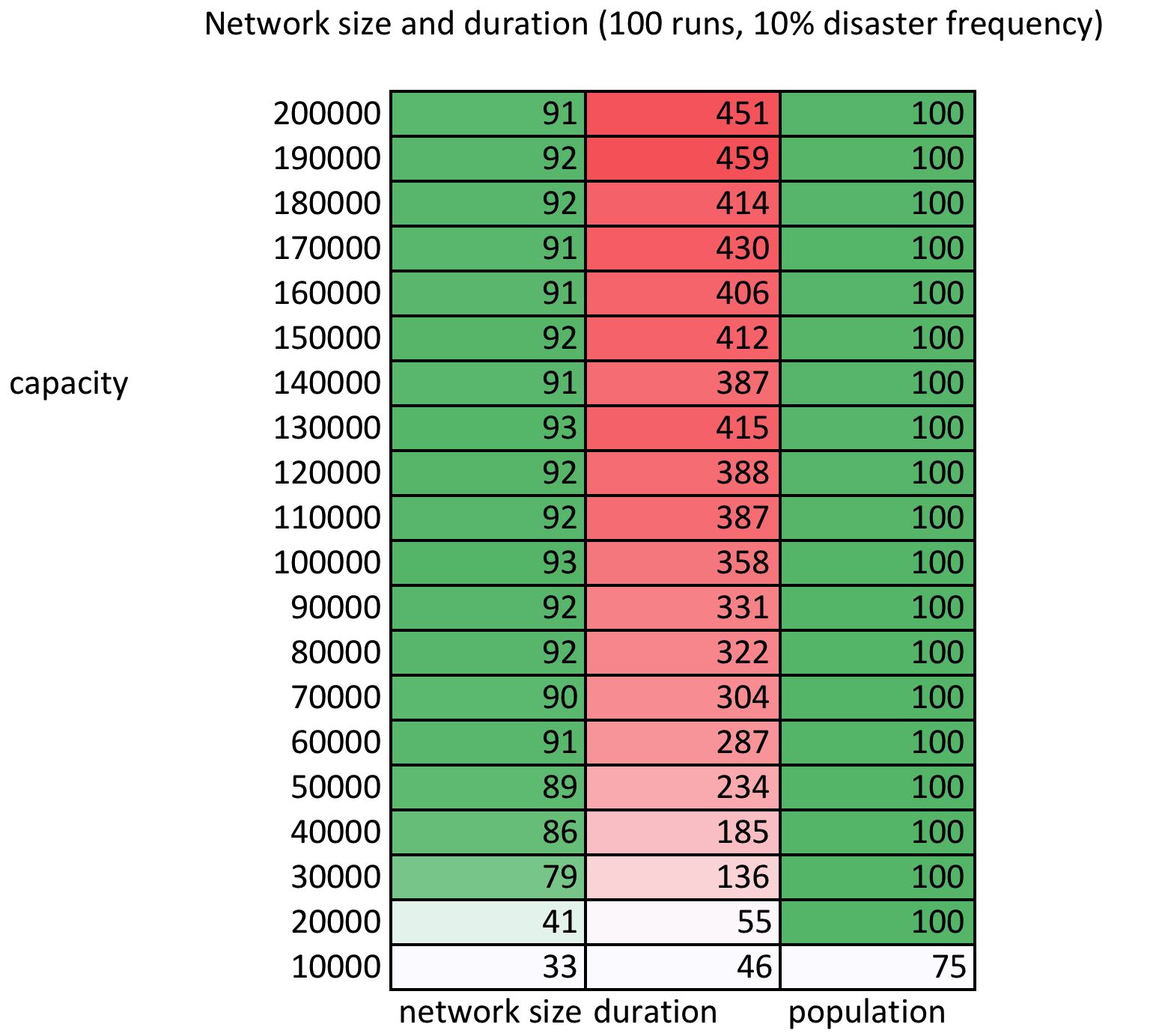


**Fig P: Network duration and size up to 200,000 carrying capacity – 10% disaster frequency – 100 runs average**


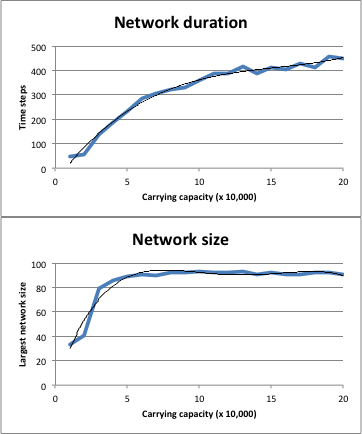


**f) Experimentation with disaster frequency range**

Disaster frequencies between 5 and 15% present a good fit with ethnographic evidence. Here we report results from tests at 1% and 20% disaster frequencies to illustrate dynamics. At 20%, household average losses per time step (disaster frequency * disaster effect) equal the growth rate. Therefore, as household herds fluctuate above and below their starting point of 60, in a short time each household will drop under 2 animals and dissolve. The result is always complete extinction of each household (see Figs Q and R). Clearly this ratio of growth to loss cannot be empirically realistic for nomadic pastoral herding over long time spans. Conversely, a 1% disaster frequency (averaging only one disaster every hundred years) is also not empirically defensible for the documented high risk of nomadic herding in Inner Asia. While a 1% disaster frequency results in the longest network durations, the size of these networks is comparatively very small (Fig S). Consequently, in this paper we focus our attentions on results from the empirically defensible range of 5 to 15% disaster frequencies coupled with a 10% growth rate.

**Fig Q: End population (out of 100 starting households), averaged from 100 runs**


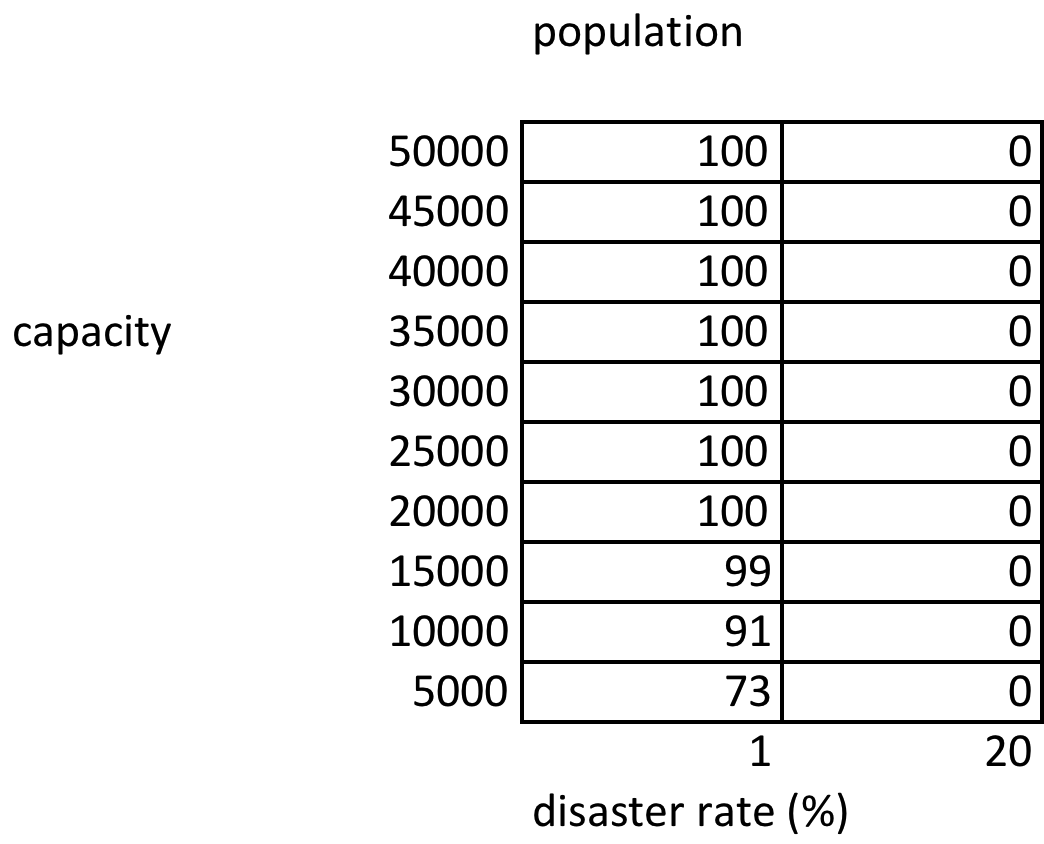


**Fig R: Average population over time from 100 runs – 50,000 carrying capacity and 20% disaster frequency**


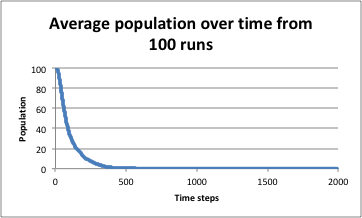


**Fig S: Largest network size and duration, averaged from 100 runs at 1% disaster frequency**


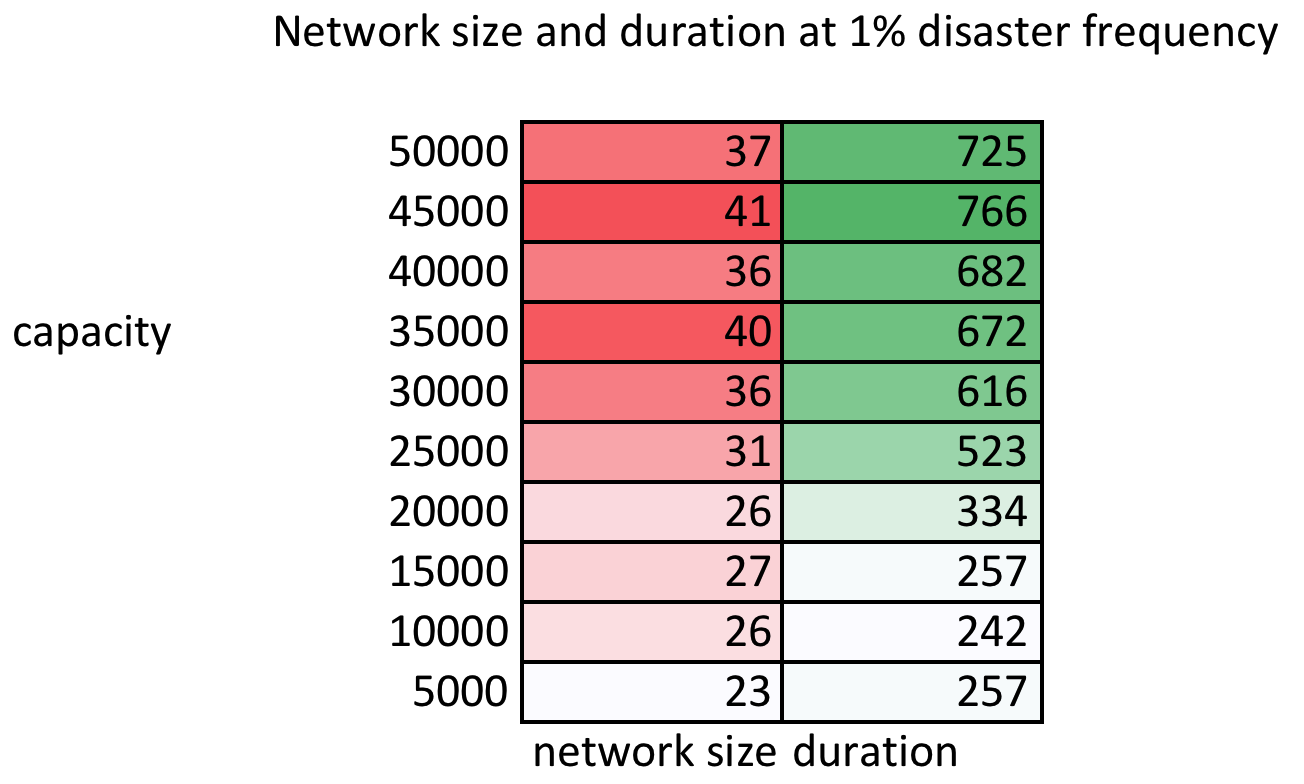


**g) Population size time series analysis**

Within our main disaster frequency test range of 5 to 15%, household deaths occur at 15% disaster frequency (due to average losses approaching average growth – see Section F in S1 file above), and at carrying capacities under 15,000 (Fig T). This latter is because the low cap on total system animals makes it difficult for the wealthiest patrons to grow their herds sufficiently to support their clients without impoverishing themselves. The time series figures on population below (Figs U and V) show that in all cases, household deaths (which can only happen when no households in the system have 800 or more animals) occur quickly. The population then stabilizes for the rest of the run, as lowering the population numbers functionally raises carrying capacity per household, such that patrons can adequately support their remaining clients.

**Fig T: End population (out of 100), averaged over 100 runs**


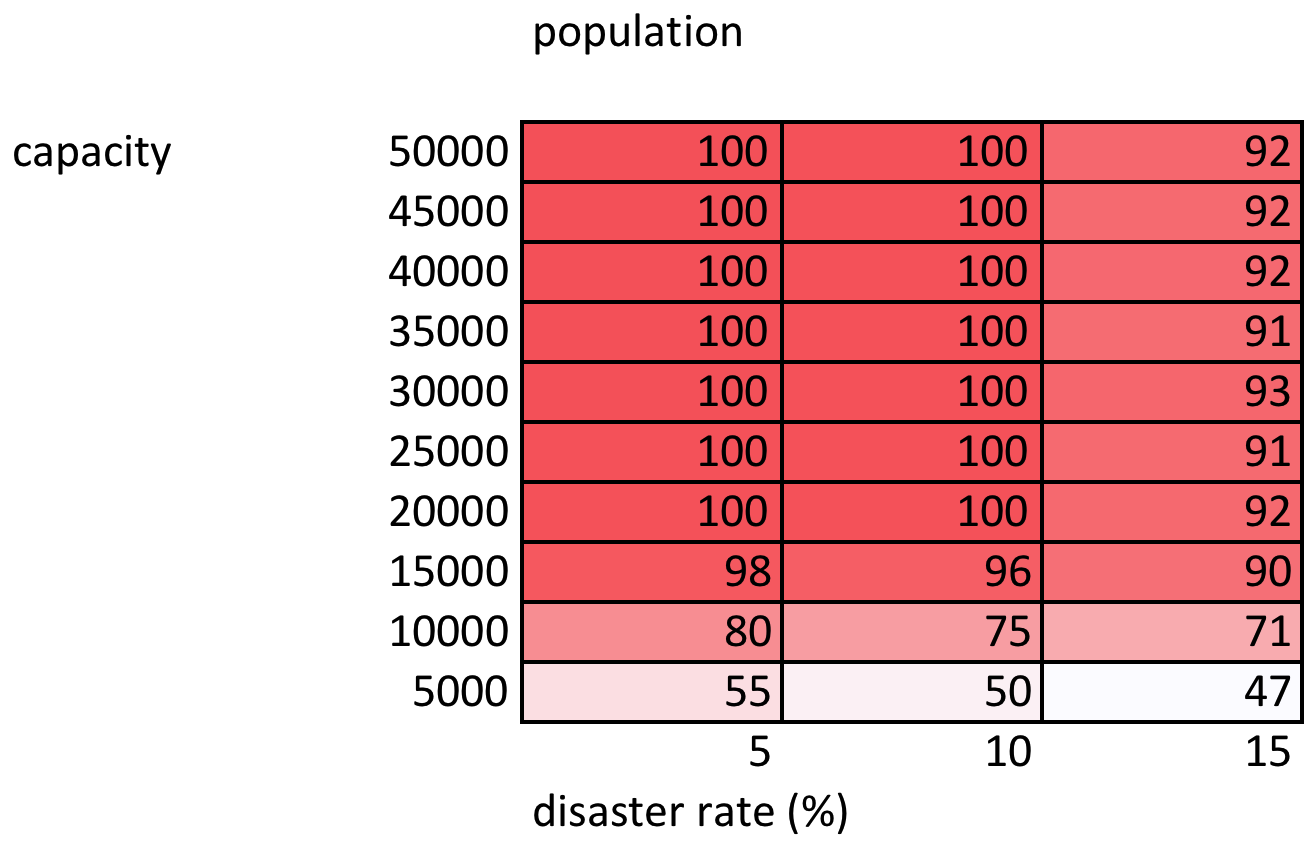


**Fig U: Population over time at 50,000 carrying capacity and 15% disaster frequency**


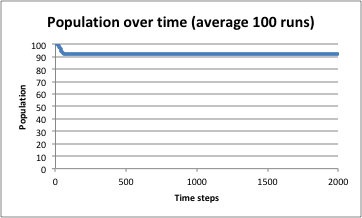


**Fig V: Population over time at 5,000 carrying capacity and 10% disaster frequency**


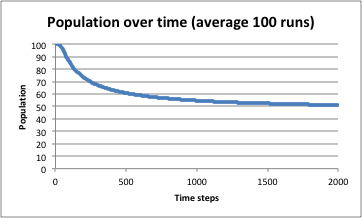


**h) Simulation animation explanation:**

Supporting information S1 Video is a video of a sample simulation run at a 50,000 animal carrying capacity and 10% disaster frequency. Households are represented by blue triangles, which turn red when that household is a patron. Links with clients are shown by an edge (line) connecting the patron to the client household. This animation illustrates the regular rise and fall of multiple patron households and their networks over the course of a 2,000 time step run. Frequently there are multiple patrons with networks on the landscape at the same time.

**Supplementary Information references:**

Lee, J. S., Filatova, T., Ligmann-Zielinska, A., Hassani-Mahmooei, B., Stonedahl, F., Lorscheid, I., ... & Parker, D. C. (2015). The complexities of agent-based modeling output analysis. *Journal of Artificial Societies and Social Simulation*, *18*(4), 4.

Lorscheid, I., Heine, B.-O. & Meyer, M. (2012). Opening the 'black box of simulations: Increased transparency and effective communication through the systematic design of experiments. *Computational and Mathematical Organization Theory*, 18, 22–62.
