# Supplementary material for: Modeling environmental variability and network formation among pastoral nomadic households: Implications for the rise of the Mongol Empire
Source: PLoS One. 2019 Oct 10;14(10):e0223677. doi: 10.1371/journal.pone.0223677 (PMC6786613; doi:10.1371/journal.pone.0223677)
Supplement: S2 File — (DOCX) [file pone.0223677.s002.docx]

NetLogo code for “Modeling environmental variability and network formation among pastoral nomadic households: implications for the rise of the Mongol Empire”

globals [

capacity

gini-index-reserve

lorenz-points

gini

leaderboard

longest-leader

urban-population

most-total-clients

most-clients

p-list

biggest-network

max-patron-counter

network-list

turtle-id-list

duration

patron-counter-list

my-duration-list

population

]

turtles-own [

herd

new-herd

herd-list

time-at-top

total-herd

patron

partner

patron?

clients

total-clients

p

patron-counter

client-counter

my-duration

]

;; setup procedures:

to setup ;; using setup button

clear-all

setup-patches

setup-turtles

set capacity 6000 ;; starting total herd size (not total carrying capacity): 100 households x 60 animals each

;;update-lorenz-and-gini

list-herds

set network-list (list)

set turtle-id-list (list)

set population (list)

reset-ticks

end

to setup-patches

ask patches [ set pcolor green ]

ask n-of num-cities patches [ set pcolor white ] ;; num-cities slider, set to 0 for chapter 3 model; set to 1 for chapter 4 model

end

to setup-turtles

create-turtles number ;; slider on interface for starting population size

ask turtles [ setxy random-xcor random-ycor ]

ask turtles [ set herd 60 ] ;; starting herd size

ask turtles [ set herd-list (list)]

ask turtles [ set time-at-top 0 ]

ask turtles [ set color blue]

end

;; runtime procedures:

to go

if ticks >= 2000 [ stop ]

grow-herd

check-disaster

check-death

stop-being-patron

stop-having-patron

find-patron

calculate-largest-network

update-lorenz-and-gini

list-herds

show-wealth

count-leadership

count-turtles

calculate-patron-counter

tick

end

to grow-herd

ask turtles [

if capacity <= capacity-level [ ;; capacity-level = interface slider representing carrying capacity

set new-herd (herd * growth-rate) ;; growth-rate = interface slider

set capacity (capacity + (new-herd - herd))

set herd (herd + (new-herd - herd))

]

ifelse show-herd?

[ set label herd ]

[ set label "" ]

]

end

to check-disaster

ask turtles [

if random 100 < disaster-rate and herd >= 2 [ ;; disaster-rate = interface slider

set new-herd (herd * disaster-effect) ;; disaster-effect = interface slider

set capacity (capacity - (herd - new-herd))

set herd (herd * disaster-effect)

]

]

end

to stop-being-patron

ask turtles with [color = red] [

if herd < 500 [

ask my-in-links [die]

set clients 0

set color blue

]

]

ask turtles with [color = blue] [

set clients 0

]

end

to stop-having-patron

ask turtles [

if patron? = 1 and patron != nobody and [color] of patron = blue [

set patron nobody

set patron? 0

]

]

end

to check-death

ask turtles [

if [herd] of self < 2 [die]

]

end

to find-patron

if any? turtles with [herd >= 800] [

ask turtles with [herd < 60] [

ifelse patron? = 1 and patron != nobody and [herd] of patron >= 800

[borrow-from-patron]

[ifelse any? other turtles in-radius radius with [herd >= 800] ;; radius = interface slider, always set high enough to cover whole grid. This slider is for future experimentation and was not a variable used in these simulations.

[select-patron]

[turtles-check-death]

]

]

]

end

to borrow-from-patron

set herd (herd + 60)

ask patron [set herd (herd - 60)]

end

to select-patron

ask my-out-links [die]

set patron? 0

choose-patron

set patron one-of other turtles in-radius radius with [color = white]

create-link-to patron

set patron? 1

set herd (herd + 60)

ask patron [set herd (herd - 60)]

ask patron [set color red]

ask patron [set clients (clients + 1)]

ask patron [set total-clients (total-clients + 1)]

end

to calculate-p

let wealth-list sort-by > [herd] of turtles in-radius radius with [herd >= 800]

let total-wealth sum [herd] of turtles in-radius radius with [herd >= 800]

ask turtles in-radius radius with [herd >= 800] [

set p precision (herd / total-wealth) 2

]

set p-list sort-by > [p] of turtles in-radius radius with [herd >= 800]

end

to choose-patron

calculate-p

while [all? turtles in-radius radius with [herd >= 800] [color != white]] [

ask other turtles in-radius radius with [herd >= 800] [

if random-float 1 <= [p] of self [

if all? other turtles in-radius radius with [herd >= 800] [color != white] [

set color white

stop

]

]

]

]

end

to turtles-check-death

if herd < 2 [die]

end

to calculate-largest-network

if count turtles >= 1 [

let count-clients sort [clients] of turtles

set network-list lput max (count-clients) network-list

if max (count-clients) > biggest-network [

set biggest-network (max count-clients)

]

ask one-of turtles with [clients = max (count-clients)][

set turtle-id-list lput self turtle-id-list

]

ask turtles [if clients = biggest-network [set my-duration my-duration + 1]]

set population lput count turtles population

]

end

to calculate-patron-counter

ask turtles with [color = red] [

set patron-counter (patron-counter + 1)

]

end

to update-lorenz-and-gini ;; to produce the lorenz points and gini index calculation necessary for graphing results

let num-people count turtles

let sorted-wealths sort [herd] of turtles ;;

let total-wealth sum sorted-wealths

let wealth-sum-so-far 0

let index 0

set gini-index-reserve 0

set lorenz-points []

repeat num-people [

set wealth-sum-so-far (wealth-sum-so-far + item index sorted-wealths)

set lorenz-points lput ((wealth-sum-so-far / total-wealth) * 100) lorenz-points

set index (index + 1)

set gini-index-reserve

gini-index-reserve +

(index / num-people) -

(wealth-sum-so-far / total-wealth)

set gini ((gini-index-reserve / count turtles) * 2)

]

end

to list-herds

ask turtles [

set herd-list lput herd herd-list

;;show (herd-list)

]

end

to show-wealth

ask turtles [

if ticks >= 1999 [

set total-herd sum herd-list

;;show total-herd

]

]

end

to count-leadership

if count turtles >= 1 [

ask turtles [

if ticks >= 0 [

set leaderboard sort [herd] of turtles

if [herd] of self = max (leaderboard) [

set time-at-top (time-at-top + 1)

]

]

]

if ticks >= 1999 [

let total-time sum [time-at-top] of turtles

;;show total-time

]

if ticks >= 1999 [

set longest-leader sort [time-at-top] of turtles

set patron-counter-list sort [patron-counter] of turtles

set my-duration-list sort [my-duration] of turtles

;;show max (longest-leader)

;;show max-one-of turtles [time-at-top]

]

]

end

to count-turtles

if count turtles >= 1 [

if ticks >= 1999 [

let turtle-count count turtles

let patron-count count turtles with [color = red]

set most-total-clients sort [total-clients] of turtles

;;show max (most-total-clients)

set most-clients sort [clients] of turtles

;;show max (most-clients)

;;show biggest-network

set max-patron-counter sort [patron-counter] of turtles

set duration 0

let index position max (network-list) network-list

let n length turtle-id-list - index

let i 1

while [(i < n) and (item (index + i) turtle-id-list = item index turtle-id-list)] [

set duration duration + 1

;;]

set i i + 1

]

set i 1

while [(i < index) and (item (index - i) turtle-id-list = item index turtle-id-list)] [

set duration duration + 1

;; ]

set i i + 1

]

]

]

end
